# Sex differences in the independent and combined effects of genomic and exposomic risks for schizophrenia on distressing psychotic experiences: insights from the ABCD study

**DOI:** 10.1007/s00737-025-01644-4

**Published:** 2026-01-06

**Authors:** Thanavadee Prachason, Angelo Arias-Magnasco, Bochao Danae Lin, Jim van Os, Bart P. F. Rutten, Lotta-Katrin Pries, Sinan Guloksuz

**Affiliations:** 1 Department of Psychiatry, Faculty of Medicine Ramathibodi Hospital, Mahidol University, Bangkok, Thailand; 2 Department of Psychiatry and Neuropsychology, School for Mental Health and Neuroscience, Maastricht University Medical Center, P.O. Box 616, Maastricht, 6200 MD The Netherlands; 3 Department of Psychiatry, UMC Utrecht Brain Centre, University Medical Centre Utrecht, Utrecht University, Utrecht, The Netherlands; 4 Department of Psychosis Studies, Institute of Psychiatry, Psychology & Neuroscience, King’s College London, London, UK; 5 Department of Psychiatry and Psychotherapy, University of Cologne, Faculty of Medicine and University Hospital Cologne, Cologne, Germany; 6 Department of Psychiatry, Yale University School of Medicine, New Haven, CT USA; 7 Department of Psychiatry, University of British Columbia, Vancouver, BC Canada; 8 Institute of Mental Health, University of British Columbia, Vancouver, BC Canada; 9 Northern Medical Program, University of Northern British Columbia, Prince George, BC Canada

**Keywords:** Sex difference, Genome, Exposome, Gene-environment interaction, Psychotic experiences, Adolescence

## Abstract

**Purpose:**

To investigate sex-dependent effects of polygenic risk (PRS-SCZ) and exposome score (ES-SCZ) for schizophrenia, both independently and jointly, on distressing psychotic experiences (PEs) in early adolescents.

**Method:**

Baseline to 3-year follow-up data of the Adolescent Brain and Cognitive Development Study (ABCD) were used. PRS-SCZ and ES-SCZ were calculated to assess cumulative genetic and environmental (childhood adversity, cannabis use, hearing impairment, and winter births) risk for schizophrenia, respectively. The primary outcome was past-month distressing PEs at the 3-year follow-up. Secondary outcomes included distressing PEs across four yearly assessments: lifetime (≥ 1 wave), repeated (≥ 2 or ≥ 3 waves), and persisting (≥ 4 waves). Sex-stratified multilevel logistic regression models were used to test the independent and joint associations of binary modes (> 75th percentile) of PRS-SCZ (PRS-SCZ_75_) and ES-SCZ (ES-SCZ_75_) on the outcomes. As sensitivity analysis, the sex-stratified analyses were repeated on a randomly selected unrelated sample, and the coefficients of males and females were compared.

**Results:**

PRS-SCZ_75_ was not associated with past-month distressing PEs in either sex but significantly associated with lifetime and repeated (≥ 2 waves) distressing PEs only in females. In both sexes, ES-SCZ_75_ was significantly associated with all PE outcomes but did not additively interact with PRS-SCZ_75_ in predicting them. Sensitivity analysis confirmed the findings and revealed a significant sex difference in the association between PRS-SCZ_75_ and lifetime distressing PEs.

**Conclusion:**

The influence of genomic risk for schizophrenia on distressing PEs might be sex-dependent, whereas that of the exposomic risk was universal in early adolescence. Further studies in larger samples are needed.

**Supplementary Information:**

The online version contains supplementary material available at 10.1007/s00737-025-01644-4.

## Article highlights

- Genomic risks for schizophrenia were associated with distressing PEs only in female adolescents.
- Environmental risks for schizophrenia were associated with distressing PEs regardless of sex in early adolescence.
- Dose-response effects of the environmental risks on PE persistence support them as potential preventive targets.
- A sex-informed approach is needed to better understand genetic and environmental contributions to psychosis expression.

**Supplementary Information:**

The online version contains supplementary material available at 10.1007/s00737-025-01644-4.

## Introduction

Psychotic experiences (PEs), including subclinical hallucinations and delusions, often emerge during childhood and adolescence (Healy et al. 2019). Early PEs not only increase the risk of later psychotic spectrum disorders (PSD) but are also associated with other severe mental health outcomes in adulthood, particularly when persisting over an extended period (Kelleher et al. 2012; Staines et al. 2023). Therefore, understanding the factors contributing to PEs in youth is essential to optimizing early intervention strategies.

The development of psychosis is influenced by gene-environment interaction (Wahbeh and Avramopoulos 2021). To quantify polygenetic background, the polygenic risk score for schizophrenia (PRS-SCZ) has been developed as a weighted sum of schizophrenia risk alleles (International Schizophrenia Consortium et al. 2009) that could predict PSD and other mental health outcomes in both clinical and general populations (Mistry et al. 2018; Pries et al. 2020). Much like genetic risks, environmental exposures influence mental health through complex and cumulative effects (Guloksuz et al. 2018). The exposome score for schizophrenia (ES-SCZ) has been developed as a weighted aggregated score of important environmental risk factors for schizophrenia and shown to predict psychosis expression from subtle to severe phenotypes (Pries et al. 2020, 2021). Furthermore, research suggests that ES-SCZ and PRS-SCZ jointly influence PSD (Pries et al. 2020) and PEs in early adolescence (Di Vincenzo et al. 2025), highlighting the intricate interplay between genetic and environmental factors in psychosis expression.

Adding further complexity to the pathoetiology of psychosis, emerging evidence highlights the pivotal role of sex in shaping the development, progression, and outcomes of PSD (Morgan et al. 2008). While prior studies suggest sex-dependent impacts of individual environmental factors on psychosis expression (Pence et al. 2022), research on sex-specific effects of exposomic risks remains limited. Notably, ES-SCZ has shown sex-specific associations with physical health, with worse outcomes in females (Paquin et al. 2023). However, its role for PEs during adolescence remains unexplored. Likewise, research on sex-specific effects of PRS-SCZ especially on PEs is scarce (Docherty et al. 2020; Mas-Bermejo et al. 2023), creating a crucial gap in our understanding of adolescent mental health.

In this study, sex differences in the independent and additive effects of PRS-SCZ and ES-SCZ on distressing PEs were investigated in a young general population cohort. Such understanding would be fundamental to personalizing early preventive strategies at a population level.

## Methods

### Participants

The ABCD Study is a multisite cohort following the development of 11,876 children in the US from early adolescence to young adulthood (Barch et al. 2018). The current study utilized data from baseline to 3-year follow-up of this cohort, Data Release 5.1 (see Acknowledgements). Inclusion criteria for this study were European descent with good-quality genotyping data and a binary sex reported at birth. Samples with incomplete data were excluded from related analyses (see Online Resource 1). The ABCD study was approved by the Centralized Institutional Review Board (IRB) of the University of California-San Diego and local research site IRBs following their IRB-approved protocols, state regulations, and local resources. Written informed consent and assent were derived from participating parents/caregivers and adolescents, respectively (Barch et al. 2018).

### Measurements

#### Psychotic experiences

PEs were self-reported by adolescents using the 21-item Prodromal Questionnaire-Brief Child Version. This scale assesses PEs (e.g., unusual thought content and perceptual abnormality) over the past month (Loewy et al. 2011). Following previous research (Karcher et al. 2022), ‘distressing PEs’ were defined as ≥ 1 PEs with a distressing score ≥ 3 of a 5-point scale. The primary outcome was distressing PEs reported at 3-year follow-up (hereafter ‘past-month distressing PEs’). As secondary outcomes, four variables indicating distressing PEs at varying persistence thresholds from baseline to 3-year follow-up were generated: distressing PEs present in ≥ 1 waves (hereafter ‘lifetime distressing PEs’), ≥ 2 waves (hereafter ‘repeating distressing PEs ≥ 2 waves’), ≥ 3 waves (hereafter ‘repeating distressing PEs ≥ 3 waves’), and all 4 waves (hereafter ‘persisting distressing PEs’).

#### Exposome score for schizophrenia

Environmental risk exposure, comprising childhood adversity (emotional and physical neglect; emotional, physical, and sexual abuse), bullying, cannabis use, winter birth, and hearing impairment, was taken from baseline to 2-year follow-up assessment and was dichotomized to indicate lifetime exposure to each risk factor (see Online Resource 1 for details). Following previous research (Pries et al. 2019), ES-SCZ was calculated by adding the nine exposures multiplied by their weighted schizophrenia risks, indicating cumulative environmental risks for schizophrenia.

#### Genotypic data

Genetic data underwent standard quality control (QC) following the RICOPILI pipeline (Lam et al. 2020) and were imputed to the TOPMed reference panel (v. R2, GRCh38). Post-imputation QC excluded variants with MAF < 1%, INFO < 0 0.9, ambiguous or multiallelic SNPs, insertion/deletions, and HWE *p* < 1 × 10^− 6^ (see Online Resource 1 for details).

#### Polygenic risk score for schizophrenia

PRS-SCZ was constructed for participants of European ancestry who passed QC (*n* = 5,656), using a Bayesian framework method with continuous shrinkage (cs) on SNP effect sizes (Ge et al. 2019) derived from the most recent schizophrenia GWAS (European subsample) (Trubetskoy et al. 2022). The 1000 Genomes Project European Sample (https://github.com/getian107/PRScs) was used as an external linkage disequilibrium reference panel. Posterior effect sizes were estimated under default PRS-cs-auto settings. PRS-SCZ was calculated in PLINK 1.9 (‘—score’ with the SUM modifier) (Purcell et al. 2007) using 742,011 variants that passed QC (see Online Resource 1 for details).

#### Statistical analysis

All analyses were conducted using Stata (version 16.1). PRS-SCZ and ES-SCZ were dichotomized at the 75th percentile for each sex separately (hereafter PRS-SCZ_75_ and ES-SCZ_75_, respectively), at which cutoff has been shown to be associated with schizophrenia case-control status (Guloksuz et al. 2019), schizotypal traits in siblings and healthy participants (Pries et al. 2020), and PEs in this population (Di Vincenzo et al. 2025). Multilevel logistic regression, accounting for study site and family structure, was used to examine the associations of PRS-SCZ_75_ and ES-SCZ_75_ with PEs. Additive interactions between PRS-SCZ_75_ and ES-SCZ_75_ were indicated by the relative excess risk due to interaction (RERI), using the delta method. A RERI > 0 indicates an interaction effect beyond the sum of genomic and exposome risk states. Sex-stratified analyses were applied for the primary outcome (past-month distressing PEs) and each secondary outcome (lifetime, repeating ≥ 2 or 3 waves, and persisting distressing PEs). Model 1 was adjusted for age, and Model 2 was further adjusted for family income and parental education. Models including PRS-SCZ_75_ were adjusted for the first ten genetic principal components (Online Resource 1). As a sensitivity analysis, we randomly selected one participant per family to create an unrelated sample and reran similar sex-stratified regression analyses. Then, sex differences in the independent and joint associations of PRS-SCZ_75_ and ES-SCZ_75_ were determined using Chow’s test (Chow 1960). Similar analyses using different percentile cutoffs for PRS-SCZ and ES-SCZ were also performed to test robustness of the findings.

## Results

This study included 2,401 females and 2,721 males with mean ages (SD) of 13 (0.7) and 12.9 (0.7) at 3-year follow-up, respectively. Sample characteristics and the prevalence of distressing PEs are shown in Table 1.

**Table 1:** Participant characteristics

Characteristics at baseline	Males (*N* = 2,721)		Females (*N* = 2,401)	
	Mean	SD	Mean	SD
Age (years)	9.95	0.63	9.91	0.63
Parental educational attainment (years)	18.2	1.7	18.2	1.7
	**n**	**%**	**n**	**%**
Family income^a^				
< $5,000	16	0.6	12	0.5
$5,000-$11,999	24	0.9	10	0.4
$12,000-$15,999	16	0.6	14	0.6
$16,000-$24,999	37	1.4	41	1.8
$25,000-$34,999	67	2.6	60	2.6
$35,000-$49,999	143	5.5	125	5.4
$50,000-$74,999	362	13.9	306	13.3
$75,000-$99,999	423	16.3	418	18.1
$100,000-$199,999	1,104	42.4	954	41.3
≥ $200,000	410	15.8	370	16.0
Lifetime exposure up to 2-year follow-up	**n**	**%**	**n**	**%**
Physical abuse	25	0.9	13	0.5
Emotional abuse	24	0.9	28	1.2
Sexual abuse	64	2.4	62	2.6
Physical neglect	541	19.9	421	17.5
Emotional neglect	67	2.5	51	2.1
Bullying	839	30.8	647	27.0
Winter birth	872	32.1	699	29.1
Hearing impairment	223	8.2	142	5.9
Cannabis use	5	0.2	3	0.1
At outcome assessment (3- year follow-up)	Mean	SD	Mean	SD
Age (years) 12.9 0.7	12.9	0.7	13.0	0.7
Distressing PEs	**n**	**%**	**n**	**%**
Past-month	209	7.7	352	14.7
Lifetime (≥ 1 wave)	850	31.2	901	37.5
Repeating ≥ 2 waves	333	12.2	408	17.0
Repeating ≥ 3 waves	130	4.8	159	6.6
Persisting (all 4 waves)	34	1.3	64	2.7

^a^ Due to missing data, N = 2,602 for males 455 and 2,310 for females

### Association of PRS-SCZ on PEs

**Fig. 1:**
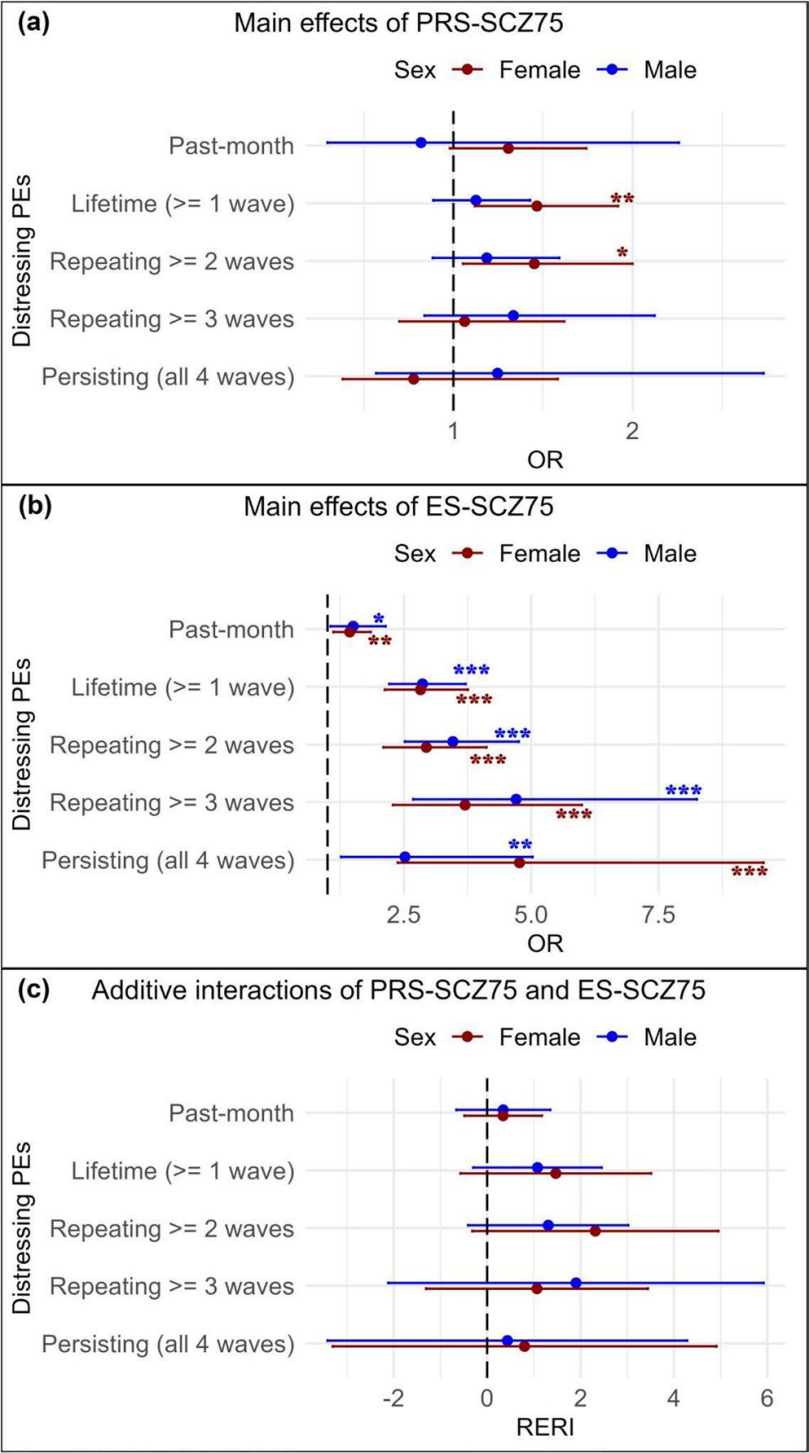
Visualization of age-adjusted main effects of PRS-SCZ_75_ (**a**) and ES-SCZ_75_ (**b**) and their joint effects (**c**) on distressing PEs in male and female adolescents (^*^ *p* <.05, ^**^ *p* <.01, ^***^ *p* <.001)

For females, PRS-SCZ_75_ was not significantly associated with past-month distressing PEs but with lifetime (OR 1.47 [95%CI 1.12, 1.92]) and repeating (≥ 2 waves) distressing PEs (OR 1.45 [95%CI 1.05, 2.00]). No significant associations were found for repeating (≥ 3 waves) and persisting distressing PEs (Table 2, Model 1, Fig. 1a). Findings from Model 2, additionally adjusted for family income and parental education, revealed similar results (Table 2).

**Table 2:** Main associations of PRS-SCZ_75_ with distressing PEs at 3-year follow-up in male and female adolescents

Distressing PEs	Males						Females					
	Model 1^a^ (*N* = 2,721)			Model 2^b^ (*N* = 2,602)			Model 1^a^ (*N* = 2,401)			Model 2^b^ (*N* = 2,310)>		
	OR	95% CI	*p*-value	OR	95% CI	*p*-value	OR	95% CI	*p*-value	OR	95% CI	*p*-value
Past-month	0.82	0.30 to 2.26	0.700	0.67	0.23 to 2.02	0.480	1.31	0.98 to 1.74	0.068	1.28	0.95 to 1.73	0.098
Lifetime (≥ 1 wave)	1.13	0.89 to 1.43	0.329	1.08	0.85 to 1.37	0.530	1.47	1.12 to 1.92	0.005^*^	1.39	1.07 to 1.81	0.015^*^
Repeating ≥ 2 waves	1.19	0.88 to 1.59	0.254	1.12	0.84 to 1.51	0.445	1.45	1.05 to 2.00	0.023^*^	1.42	1.02 to 1.97	0.038^*^
≥ 3 waves	1.33	0.84 to 2.12	0.223	1.31	0.80 to 2.16	0.282	1.06	0.70 to 1.62	0.776	0.99	0.64 to 1.53	0.968
Persisting (all 4 waves)	1.25	0.57 to 2.73	0.582	1.19	0.54 to 2.64	0.665	0.78	0.38 to 1.58	0.488	0.62	0.27 to 1.40	0.252

PEs, psychotic experiences; OR, odds ratio; CI, confidence interval

^a^ Model 1: Adjusted for age; ^b^ Model 2: Adjusted for age, family income, and parental education

^*^ *p*-values < 0.05

For males, PRS-SCZ_75_ was not significantly associated with past-month distressing PEs or any secondary outcomes (Table 2, Model 1, Fig. 1a). The results from Model 2 further supported these findings (Table 2).

Sensitivity analyses confirmed differential associations between PRS-SCZ_75_ and lifetime and repeating (≥ 2 waves) distressing PEs in females but not males (Online Resource 2). Follow-up analyses indicated significant sex difference for lifetime distressing PEs (≥ 1 wave) across both adjusted models (*p* <.001). No other significant sex differences were observed.

### Association of ES-SCZ on PEs

For females, ES-SCZ_75_ was significantly associated with past-month distressing PEs (OR 1.44 [95%CI 1.12, 1.85]) and all secondary outcomes (lifetime: OR 2.83 [95%CI 2.13, 3.76]; repeating ≥ 2 waves: OR 2.94 [95%CI 2.10, 4.12]; repeating ≥ 3 waves: OR 3.70 [95%CI 2.29, 5.99]; and persisting distressing PEs: OR 4.77 [95%CI 2.38, 9.56]). As shown in Fig. 1b, the ORs of ES-SCZ_75_ generally increase for more PE persistence. The results from Model 2 further supported these findings (Table 3).

**Table 3:** Main associations of ES-SCZ_75_ at 2-year follow-up with distressing PEs at 3-year follow-up in male and female adolescents

Distressing PEs	Males						Females					
	Model 1^a^ *N* = 2,721)			Model 2^b^ (*N* = 2,602)			Model 1^a^ (*N* = 2,**401)**			Model 2^b^ (*N* = 2,310)		
	OR	95% CI	*p*-value	OR	95% CI	*p*-value	OR	95% CI	*p*-value	OR	95% CI	*p*-value
Past-month^c^	1.50	1.06 to 2.14	0.023^*^	1.47	1.00 to 2.15	0.050	1.44	1.12 to 1.85	0.005^*^	1.35	1.03 to 1.76	0.031^*^
Lifetime (≥ 1 wave)	2.87	2.21 to 3.71	< 0.001^*^	2.70	2.09 to 3.49	< 0.001^*^	2.83	2.13 to 3.76	< 0.001^*^	2.48	1.87 to 3.28	< 0.001^*^
Repeating ≥ 2 waves	3.46	2.52 to 4.75	< 0.001^*^	3.23	2.38 to 4.39	< 0.001^*^	2.94	2.10 to 4.12	< 0.001^*^	2.62	1.87 to 3.66	< 0.001^*^
Repeating ≥ 3 waves	4.70	2.68 to 8.25	< 0.001^*^	4.35	2.39 to 7.92	< 0.001^*^	3.70	2.29 to 5.99	< 0.001^*^	3.15	1.97 to 5.04	< 0.001^*^
Persisting (all 4 waves)	2.52	1.27 to 5.03	0.009^*^	2.41	1.16 to 5.03	0.019^*^	4.77	2.38 to 9.56	< 0.001^*^	4.08	2.01 to 8.29	< 0.001^*^

PEs, psychotic experiences; OR, odds ratio; CI, confidence interval

^a^ Model 1: Adjusted for age; ^b^ Model 2: Adjusted for age, family income, and parental education

^c^ Additionally adjusted for distressing PEs up to 2-year-follow-up

^*^ *p*-values < 0.05

For males, ES-SCZ_75_ was significantly associated with past-month distressing PEs (OR 1.50 [95%CI 1.06, 2.14]) and all secondary outcomes (lifetime: OR 2.87 [95%CI 2.21, 3.71]; repeating ≥ 2 waves: OR 3.46 [95%CI 2.52, 4.75]; repeating ≥ 3 waves: OR 4.70 [95%CI 2.68, 8.25]; and persisting distressing PEs: OR 2.52 [95%CI 1.27, 5.03]). As shown in Fig. 1b, the ORs of ES-SCZ_75_ generally increase for more PE persistence, except for persisting (4 waves) distressing PEs. The results from Model 2 further supported these findings (Table 3).

Sensitivity analyses similarly revealed increasing ORs for ES-SCZ_75_ for more PE persistence in females and males. No significant sex differences in ORs of ES-SCZ_75_ were observed for any PE definitions (Online Resource 2).

###  Joint interaction of PRS-SCZ and ES-SCZ on PEs

When considered jointly, the isolated genetic risk state (PRS-SCZ_75_ = 1 & ES-SCZ_75_ = 0) was not associated with past-month distressing PEs in either females (Table 4) or males (Table 5). However, the isolated exposomic risk state (PRS-SCZ_75_ = 0 & ES-SCZ_75_ = 1) significantly increased the odds of having past-month distressing PEs in females (OR 1.37 [95% CI 1.02, 1.85]) but not males (OR 1.45 [95% CI 0.98, 2.16], Table 5). Similarly, the combined risk state (PRS-SCZ_75_ = 1 & ES-SCZ_75_ = 1) was associated with past-month distressing PEs significantly in females (OR 1.82 [95% CI 1.19, 2.77]) but not males (OR 1.68 [95% CI 0.97, 2.90], Table 5). The combined effect did not significantly differ from the sum of the ORs of having either risk state alone, indicating a null additive interaction between PRS-SCZ_75_ and ES-SCZ_75_ in either sex (Tables 4 and 5; Fig. 1c).

**Table 4:** Joint associations of PRS-SCZ_75_ and ES-SCZ_75_ at 2-year follow-up with distressing PEs at 3-year follow-up in female adolescents

Distressing PEs		Model 1^a^ (*N* = 2,401)			Model 2^b^ (*N* = 2,310)		
		PRS-SCZ_75_ = 0 OR (95% CI)	PRS-SCZ_75_ = 1 OR (95% CI)	RERI (95% CI)	PRS-SCZ_75_ = 0 OR (95% CI)	PRS-SCZ_75_ = 1 OR (95% CI)	RERI (95% CI)
Past-month^c^ (3-year follow-up)	ES-SCZ_75_ = 0	1.0	1.10 (0.79 to 1.55) *p* =.568	0.34 (−0.49 to 1.17) *p* =.423	1.0	1.17 (0.83 to 1.66) *p* =.378	0.13 (−0.69 to 0.95) *p* =.761
	ES-SCZ_75_ = 1	1.37 (1.02 to 1.85) *p *=.036^*^	1.82 (1.19 to 2.77) *p* =.006^*^		1.34 (0.98 to 1.82) *p* =.066	1.64 (1.04 to 2.58) *p* =.034^*^	
Lifetime (≥ 1 wave)	ES-SCZ_75_ = 0	1.0	1.39 (1.02 to 1.89) *p* =.037^*^	1.47 (−0.57 to 3.51) *p* =.158	1.0	1.32 (0.97 to 1.79) *p* =.073	1.19 (−0.57 to 2.94) *p* =.185
	ES-SCZ_75_ = 1	2.57 (1.90 to 3.49) *p* <.001^*^	4.43 (2.73 to 7.20) *p* <.001^*^		2.28 (1.69 to 3.09) *p* <.001^*^	3.79 (2.34 to 6.14) *p* <.001^*^	
Repeating (≥ 2 waves)	ES-SCZ_75_ = 0	1.0	1.23 (0.83 to 1.82) *p* =.299	2.31 (−0.32 to 4.95) *p* =.085	1.0	1.21 (0.81 to 1.81) *p* =.343	2.04 (−0.37 to 4.46) *p* =.098
	ES-SCZ_75_ = 1	2.52 (1.75 to 3.62) *p* <.001^*^	5.06 (2.87 to 8.94) *p* <.001^*^		2.28 (1.58 to 3.29) *p* <.001^*^	4.53 (2.54 to 8.08) *p* <.001^*^	
Repeating (≥ 3 waves)	ES-SCZ_75_ = 0	1.0	0.90 (0.51 to 1.61) *p* =.727	1.07 (−1.31 to 3.44) *p* =.379	1.0	0.84 (0.47 to 1.52) *p* = 565	0.89 (−1.19 to 2.97) *p* =.403
	ES-SCZ_75_ = 1	3.01 (1.92 to 4.73) *p* <.001^*^	3.98 (2.07 to 7.65) *p* <.001^*^		2.64 (1.68 to 4.14) *p* <.001^*^	3.36 (1.74 to 6.51) *p* <.001^*^	
Persisting (all 4 waves)	ES-SCZ_75_ = 0	1.0	0.52 (0.16 to 1.68) *p* =.275	0.80 (−3.31 to 4.91) *p* =.703	1.0	0.51 (0.15 to 1.75) *p* =.288	−0.29 (−4.04 to 3.47) *p* =.881
	ES-SCZ_75_ = 1	4.19 (1.99 to 8.81) *p* <.001^*^	4.51 (1.60 to 12.8) *p* =.004^*^		4.04 (1.76 to 9.26) *p* =.001^*^	3.27 (1.03 to 10.4) *p* =.045^*^	

PEs, psychotic experiences; OR, odds ratio; CI, confidence interval

^a^ Model 1: Adjusted for age; ^b^ Model 2: Adjusted for age, family income, and parental education

^c^ Additionally adjusted for distressing PEs up to 2-year-follow-up

^*^ *p*-values < 0.05

**Table 5:** Joint associations of PRS-SCZ_75_ and ES-SCZ_75_ at 2-year follow-up with distressing PEs at 3-year follow-up in male adolescents

Distressing PEs		Model 1^a^ (*N* = 2,721)			Model 2^b^ (*N* = 2,602)		
		PRS-SCZ_75_ = 0 OR (95% CI)	PRS-SCZ_75_ = 1 OR (95% CI)	RERI (95% CI)	PRS-SCZ_75_ = 0 OR (95% CI)	PRS-SCZ_75_ = 1 OR (95% CI)	RERI (95% CI)
Past-month^c^ (3-year follow-up)	ES-SCZ_75_ = 0	1.0	0.88 (0.52 to 1.49) *p* =.638	0.34 (−0.66 to 1.35) *p* =.503	1.0	0.82 (0.46 to 1.44) *p* =.485	0.37 (−0.66 to 1.40) *p* =.482
	ES-SCZ_75_ = 1	1.45 (0.98 to 2.16) *p* =.065	1.68 (0.97 to 2.90) *p* =.063		1.39 (0.91 to 2.14) *p* =.129	1.58 (0.87 to 2.87) *p* =.133	
Lifetime (≥ 1 wave)	ES-SCZ_75_ = 0	1.0	0.98 (0.72 to 1.32) *p* =.880	1.08 (−0.30 to 2.45) *p* =.125	1.0	0.94 (0.69 to 1.26) *p* =.667	0.91 (−0.34 to 2.16) *p* =.153
	ES-SCZ_75_ = 1	2.55 (1.93 to 3.37) *p* <.001^*^	3.61 (2.41 to 5.41) *p* <.001^*^		2.42 (1.83 to 3.19) *p* <.001^*^	3.27 (2.18 to 4.89) *p* <.001^*^	
Repeating (≥ 2 waves)	ES-SCZ_75_ = 0	1.0	0.97 (0.64 to 1.47) *p* =.882	1.31 (−0.41 to 3.02) *p* =.136	1.0	0.91 (0.60 to 1.40) *p* =.675	1.11 (−0.43 to 2.66) *p* =.157
	ES-SCZ_75_ = 1	3.08 (2.18 to 4.35) *p* <.001^*^	4.35 (2.79 to 6.78) *p* <.001^*^		2.90 (2.07 to 4.07) *p* <.001^*^	3.93 (2.55 to 6.05) *p* <.001^*^	
Repeating (≥ 3 waves)	ES-SCZ_75_ = 0	1.0	1.16 (0.57 to 2.36) *p* =.673	1.90 (−2.12 to 5.92) *p* =.354	1.0	1.10 (0.52 to 2.32) *p* = 803	1.91 (−2.05 to 5.86) *p* =.345
	ES-SCZ_75_ = 1	4.39 (2.38 to 8.08) *p* <.001^*^	6.45 (2.95 to 14.1) *p* <.001^*^		3.94 (2.08 to 7.49) *p* <.001^*^	5.95 (2.61 to 13.6) *p* <.001^*^	
Persisting (all 4 waves)	ES-SCZ_75_ = 0	1.0	1.32 (0.44 to 3.93) *p* =.615	0.43 (−3.42 to 4.28) *p* =.826	1.0	1.28 (0.43 to 3.83) *p* =.658	0.27 (−3.31 to 3.86) *p* =.881
	ES-SCZ_75_ = 1	2.74 (1.13 to 6.66) *p* =.026^*^	3.49 (1.14 to 10.7) *p* =.028^*^		2.60 (1.05 to 6.44) *p* =.038^*^	3.16 (1.01 to 9.91) *p* =.049^*^	

PEs, psychotic experiences; OR, odds ratio; CI, confidence interval

^a^ Model 1: Adjusted for age; ^b^ Model 2: Adjusted for age, family income, and parental education

^c^ Additionally adjusted for distressing PEs up to 2-year-follow-up

^*^ *p*-values < 0.05

For secondary outcomes, the isolated genetic risk state was significantly associated with lifetime distressing PEs (OR 1.39 [95% CI 1.02, 1.89]) only in females. No significant associations were observed for other secondary outcomes in either sex. The isolated exposomic risk state and the combined risk state were associated with all secondary outcomes in both sexes (Tables 4 and 5). However, no significant additive interactions were detected (Tables 4 and 5; Fig. 1c). These findings were consistent in both adjusted models (Tables 4 and 5) and were confirmed by sensitivity analyses (Online Resource 2).

## Discussion

This study investigated the joint and independent associations of polygenic and exposomic liabilities for schizophrenia with distressing PEs and their persistence in male and female adolescents. PRS-SCZ_75_ was significantly associated with lifetime (≥ 1 wave) and repeating (≥ 2 wave) distressing PEs in females but not with any PE definitions in males. ES-SCZ_75_ was associated with all PE definitions, with a trend of higher ORs for more PE persistence in both sexes. No significant additive interactions between PRS-SCZ_75_ and ES-SCZ_75_ were observed for any PE definitions.

Our finding that PRS-SCZ was significantly associated with distressing PEs only in females suggests a sex-dependent influence of genetic risks on subclinical psychosis in early adolescence. This contradicts evidence from adults showing more dominant PRS-SCZ influences on psychosis-related phenotypes, including poor cognitive performance and corresponding brain function, in males (Koch et al. 2021, 2022). Combined with poorer cognitive function and more pronounced negative symptoms reported in males with schizophrenia (Giordano et al. 2021), such evidence implies that males are more susceptible to neurocognitive impairments, particularly under schizophrenia genetic risks. Familial aggregation and CNV studies, however, indicate that females with schizophrenia carry a higher genetic risk burden than males (Goldstein et al. 1990; Han et al. 2016). This pattern aligns with the ‘female protective effect’ hypothesis, which posits that females are inherently more resilient to certain neurodevelopmental and psychiatric conditions, like schizophrenia, disproportionately affecting males. Therefore, females may require a greater genetic load to surpass the threshold for developing psychosis-related phenotypes (Jacquemont et al. 2014; Robinson et al. 2013). Under this framework, our finding on female-specific association between PRS-SCZ and distressing PEs might be an indirect signal of this protective effect rather than evidence of a stronger genetic influence in females. Specifically, if males are more vulnerable to schizophrenia-related traits even at lower genetic risk levels, their psychosis-related phenotypes may emerge through other phenotypes (cognitive deficits or negative symptoms) rather than distressing PEs. In contrast, the emergence of distressing PEs in females might indicate that only those with a higher schizophrenia genetic load surpass the threshold for experiencing such symptoms. Interestingly, some studies reported a male-specific association of PRS-SCZ with trait-like psychosis-related phenotypes, such as schizotypy and negative symptoms, but not with state-like positive symptoms such as PEs (Docherty et al. 2020; Mas-Bermejo et al. 2024). Combined with our findings, this suggests that schizophrenia genetic risks may have sex-dependent effects, with males more likely to manifest stable, trait-like phenotypes (e.g., schizotypy, negative symptoms, and cognitive deficits), while females may be more prone to developing transient, state-like symptoms such as distressing PEs. However, due to methodological differences among studies (e.g., age ranges, genetic risk markers, and study design), future research is still needed to validate our proposed hypothesis.

From a transdiagnostic perspective, it is important to note that distressing PEs are not specific to psychotic disorders. A meta-analysis has shown that the pooled OR of PEs for affective disorders (3.83) in childhood and adolescence is comparable to that for psychotic disorders (3.96) (Healy et al. 2019). Given that female preponderance in depression emerges around age 12 (Salk et al. 2017), the female-specific PRS-SCZ association at age 13 in our study may reflect a general increase in mental ill-health rather than an association specific to PEs. Moreover, prior studies (Stainton et al. 2021; Zammit et al. 2013), like ours, found that female adolescents report higher levels of distress and recurring PEs than males, rendering the analytical power for females stronger than males. Interestingly, evidence from a general population sample reported a higher rate of positive PEs that was confounded by depressive symptoms in females (Maric et al. 2003). Therefore, large prospective studies assessing PEs and associated psychopathology across development are needed to clarify these findings.

Relative to PRS-SCZ, sex-stratified analyses of the influences of ES-SCZ are even more scarce. In this study, no statistically significant sex differences were detected for any outcomes. However, the ORs for persisting distressing PEs (4 waves) appeared almost double in females. Indeed, a stronger influence of ES-SCZ in females was reported for physical health, suggesting a greater sensitivity to the environmental insults composing ES-SCZ among females (Paquin et al. 2023). Interestingly, we consistently observed significant associations of ES-SCZ, but not PRS-SCZ, with all outcomes and a dose-response relationship with PE persistence in both sexes, highlighting a universally more dominant impact of environmental risk than genetic risk for schizophrenia on distressing PEs. Concordantly, evidence from a large male schizophrenia cohort showed a substantial impact of cumulative environmental risk, but not schizophrenia polygenic risk, on the age at schizophrenia onset (Stepniak et al. 2014). However, limited research on sex differences calls for more comparative analysis of genetic and environmental contributions to confirm our findings.

Apart from the independent associations, prior evidence has shown that the influences of genomic and exposomic risk for schizophrenia are synergistic (Pries et al. 2020). Our prior unstratified analyses of the ABCD dataset also found significant additive interactions between ES-SCZ_75_ and PRS-SCZ_75_ for distressing PEs recurring in ≥ 1–2 waves (Di Vincenzo et al. 2025). However, the current sex-stratified analyses, halving the sample size, could not detect significant additive interactions, although the ORs of the combined risk state tended to be greater than either risk alone for most outcomes (Tables 4 and 5). Considering that the CIs of the RERIs are wide and the observed RERIs are above zero in both sexes (see Fig. 1c), the non-significant additive interaction could reflect underpower of our sex-stratified analysis.

## Strengths and limitations

To our knowledge, this is the first study to examine sex-dependent influences of an aggregated environmental risk score for schizophrenia, independently and with polygenic risk, in early adolescents. Focusing on distressing and persistent PEs enhances the clinical relevance of our findings, as both are key predictors of poor outcomes (Karcher et al. 2020; Staines et al. 2023). Nevertheless, the findings should be considered in light of limitations.

First, we restricted the analysis to European subsample to ensure good performance of PRS-SCZ based on recent GWAS, limiting generalizability to non-European populations. Second, PEs in this dataset were self-reported, potentially leading to false positives compared to interview-based assessments (Staines et al. 2023). However, prior evidence has shown that non-validated self-reported PEs are significantly associated with psychopathology similar to clinically validated PEs and attenuated psychosis syndrome (Moriyama et al. 2019). Furthermore, even false-positive self-reported PEs appear to be associated with later mental health problems (van der Steen et al. 2019), supporting the clinical significance of our findings. Third, while genomic risks always precede outcomes, exposomic risks may have a bidirectional relationship with distressing PEs, potentially inflating associations. To mitigate this, we adjusted for prior-wave distressing PEs in past-month analyses, reducing the likelihood of overestimating ES-SCZ effects. However, this approach could not be applied to secondary outcomes, leaving the possibility of reverse causality or residual confounding for the analysis of ES-SCZ. Fourth, other known environmental risks for psychosis, like obstetric complications, were not included in our ES-SCZ and could be examined in future exposome studies. However, such information is rarely collected prospectively in most cohorts and remains exceedingly difficult to ascertain retrospectively without comprehensive birth registry data. Lastly, our sex-stratified analyses reduced the sample size, possibly causing underpowered analyses for low-prevalent outcomes (e.g., persisting distressing PEs) or potentially weak associations (e.g., interaction effects). This might be particularly relevant for males, given their lower prevalence of distressing PEs.

## Conclusion

Our findings on the association of PRS-SCZ and distressing PEs only in females underscore the importance of a sex-sensitive approach in studying genetic contributions to subclinical psychosis. Dose-response effects of ES-SCZ on PE persistence in both sexes highlight environmental risks as universal targets to prevent PEs and associated psychopathology in early adolescence.

## Supplementary Information

Below is the link to the electronic supplementary material.

Supplementary Material 1 (DOCX 206 KB)

## Data Availability

Data used in the preparation of this article were obtained from publicly available data from the Adolescent Brain Cognitive Development Study (https://abcdstudy.org), held in the National Institute of Mental Health Data Archive.
