# Supplementary material for: Sex differences in the independent and combined effects of genomic and exposomic risks for schizophrenia on distressing psychotic experiences: insights from the ABCD study
Source: Arch Womens Ment Health. 2026 Jan 6;29(1):7. doi: 10.1007/s00737-025-01644-4 (PMC12775040; doi:10.1007/s00737-025-01644-4)
Supplement: Supplementary file 1 — Supplementary Material 1 (DOCX 206 KB) [file 737_2025_1644_MOESM1_ESM.docx]

September 25, 2025

Editorial Office of Archives of Women’s Mental Health

Dear Prof. Anita Riecher-Rösslers,

We are very glad that you and the reviewers saw the value in our manuscript. We are grateful to you for allowing us the opportunity to further improve the manuscript, and we thank the reviewers for the constructive feedback that provided guidance in this process. We have itemized the comments and indicated below the response to each, along with verbatim changes provided as part of our responses. Briefly, the abstract and the main text were adjusted following the reviewers’ suggestions, yet complying with the word limit. We also performed sensitivity analyses using different cutoffs for PRS-SCZ and ES-SCZ to test the robustness of our findings and provided the results in the Online Resource 2. All changes are highlighted in the manuscript.

We hope that the manuscript is now acceptable in its revised form and look forward to your and the reviewers’ responses.

Sincerely,

Thanavadee Prachason, Lotta-Katrin Pries, Sinan Guloksuz

**Reviewer #1:**

In this article entitled "Sex Differences in the Independent and Combined Effects of Genomics and Exposomic Risks for Schizophrenia on Distressing Psychotic Experiences: Insight from the ABCD Study", the authors presented a clear analysis of genetic and non-genetic contributions to schizophrenia on distressing psychotic experiences (PEs), suggesting a sex-dependent genetic effects together with a all-sex environmental effects in adolescent.

The authors clearly presented their work, sometimes omitting details in the main text which might be retrieved in the Supplementary data. The introduction is well presented and easy to get and put in focus the research questions. Methods might be revisited, moving some information from the Supplementary to the main text. Results are appreciable and understandable, and might benefit from little revisions. Finally, the Discussion has been well performed and put all the results together, opening for future research.

Thank you very much for your positive feedback. We have revised the manuscript in accordance with your valuable suggestions.

**Major points**
**1.1** At first, regarding the 75th percentile dichotomization/binarization. Did the authors performed the percentile division of PRS-SCZ and ES-SCZ on the whole population or did they take into account the different sexes? Given the obtained results (with significance towards female on a genetic basis), a percentile calculation on male- and female-only samples might be worth to be explored, providing additional results on sex-specific genetic liability.

**R1.1:** Thank you for your careful inspection. We dichotomized PRS-SCZ and ES-SCZ for each sex separately. We have now clarified this important detail in the method section, as shown:

*“Following prior research(Di Vincenzo et al. 2025), PRS-SCZ and ES-SCZ were dichotomized at the 75th percentile for each sex separately (hereafter PRS-SCZ_75_ and ES-SCZ_75_, respectively).”*

**1.2** At second, given the two tested models, would the authors draw some conclusion regarding corrections and possible confounding factor effects? I think that drawing consideration from their models (which seem to be concordant) might improve their Discussion (in which this point is still present).

**R1.2:** Thank you for your thoughtful suggestion. We agree with the reviewer that the findings that the models remain statistically significant after correction are relevant and have now further elaborated on these in the discussion. We have added the following sentence:

*“The findings remained consistent when controlling for family income and parental education, precluding confounding from socioeconomic background.”*

Overall, the authors presented a good manuscript. Considering the above comments and the below list of minor issues, I would suggest major revision for this manuscript.

**1.3 Minor points**
**1.3.1** General:
   * Some typos are present, e.g. missing space in final character of words and bracket for reference (like "this word(this ref)" should be "this word (this ref)."
**R1.3.1:** Thank you for your feedback. We have carefully checked and added missing spaces throughout the manuscript as suggested.

**1.3.2** Abstract:
   * Might benefit of a short introduction
   * The results might be rewritten since they shift across concepts back and forth multiple times.

**R1.3.2:** Thank you for your constructive comments. Unfortunately, we could not add an introduction due to the abstract structure and a strict word limit (250) following the journal’s guidelines. In accordance with the reviewer’s suggestion, we have revised the results as follows:

*“Results: PRS-SCZ_75_ was not associated with past-month distressing PEs in either sex but significantly associated with lifetime and repeated (≥2 waves) distressing PEs only in females. In both sexes, ES-SCZ_75_ was significantly associated with all PE outcomes but did not additively interact with PRS-SCZ_75_ in predicting them. Sensitivity analysis confirmed the findings and revealed a significant sex difference in the association between PRS-SCZ_75_ and lifetime distressing PEs.”*

Introduction:
**1.3.3**   * At line 40, "quantify polygenetic risk, the polygenic risk" might be a possible confounding source; hence, either "polygenetic risk" might be changed in "polygenetic background" (or similar) or the sentence might be worth to be rephrased.

**R1.3.3:** Thank you for your thoughtful suggestion. We have replaced “polygenetic risk” with “polygenetic background” as suggested.

**1.3.4*** At lines 46-47, among the environmental causes, obstetric complications (OCs) are not mentioned. Do the authors think this is worth to be mentioned and/or do the authors have this information in their data?

**R1.3.4:** We appreciate your careful observation and agree that obstetric complications (OCs) are an important environmental risk factor. As noted in lines 46–47, we introduced the exposome score developed by our research group, which does not include OCs due to the unavailability of this variable in the training and validation datasets (Pries et al. 2019). For this reason, OCs were not mentioned in the introduction. In line with your comment, we have now acknowledged this limitation in the discussion as follows:

*“Fourth, other known environmental risks for psychosis, like obstetric complications, were not included in our ES-SCZ and could be examined in future exposome studies.* *However, such information is rarely collected prospectively in most cohorts and remains exceedingly difficult to ascertain retrospectively without comprehensive birth registry data.”*

 **1.3.5** * At lines 59-60, the aims of this study might be worth to be expanded a little

**R1.3.5:** Thank you for your kind suggestion. Due to space limitations, we could only briefly expand the aim of this study as follows:

*“In this study, sex differences in the independent and additive effects of PRS-SCZ and ES-SCZ on distressing PEs were investigated in a young general population cohort. Such understanding would be fundamental to personalizing early preventive strategies at a population level. ”*

 **1.3.6** Methods:

* The authors should report the parameters to assess good quality genotyped data; moreover, given the limitation of genotyped only data and following what was reported in the supplementary material, the usage of genotype imputation needs to be address in the main manuscript text?
  * At line 97, genetic and sample quality controls should be specified

**R1.3.6:** Thank you for your insightful feedback. We have now described the process of quality control of the genotyped data and genotype imputation as shown:

*“**Genetic data underwent standard quality control (QC) following the RICOPILI pipeline (Lam et al. 2020) and were imputed to the TOPMed reference panel (v. R2, GRCh38). Post-imputation QC excluded variants with MAF < 1%, INFO <0 .9, ambiguous or multiallelic SNPs, insertion/deletions, and HWE p < 1x10^-6^(see Online Resource 1 for details).”*

**1.3.7**   * Did the authors remove the Finnish population from the linkage map of the 1000 Genomes Project or do they think it does not represent a concern?.

**R1.3.7:** We thank the reviewer for raising this important point. We did not exclude Finnish individuals from the 1000 Genomes Project reference panel, as this dataset was used solely for ancestry derivation at the super-population level (EUR= 5,656, AFR=1,195, AMR=8,352 participants). Principal components were computed on LD-pruned SNPs from the combined ABCD and 1000 Genomes dataset, which minimizes the impact of long-range linkage disequilibrium patterns characteristic of Finnish ancestry. Moreover, imputation was conducted using the TOPMed reference panel (v. R2, GRCh38), not the 1000 Genomes Project. We therefore consider the inclusion of Finnish individuals in the 1000 Genomes reference to not represent a concern for our analyses.

**1.3.8**  * The PLINK1.9 tool should be referenced

**R1.3.8:** We have added the reference as shown:

*“PRS-SCZ was then constructed based on 742,011 variants that pass quality control, using ‘--score’ function and the SUM modifier in PLINK1.9 (Purcell et al. 2007).”*

**1.3.9**  * Moreover, PRS-SCZ standardization should be reported

**R1.3.9:** Thank you for your kind suggestion. Since we dichotomized PRS-SCZ before performing all statistical analyses, we did not standardize PRS-SCZ; therefore, the sentence about standardization has been removed from the manuscript.

**1.3.10** Results:
   * The authors should briefly introduce the samples under investigation, thus expanding line 122

**R1.3.10:** Thank you for your thoughtful suggestion. We have briefly introduced the study sample as follows:

*“**This study included 2,401* *females and 2,721 males with mean ages (SD) of 13 (0.7) and 12.9 (0.7) at 3-year follow-up, respectively. Sample characteristics and the prevalence of distressing PEs are shown in Table 1.”*

**1.3.11**  * The authors perform analysis on two models; however, sample splitting and numerosity seem to me quite unclear. Can the authors provide an explanation of the sample involved?

**R1.3.11:** Thank you for your careful inspection. The total number of male and female adolescents of European ancestry was 2,984 and 2,665, respectively. The number of participants with no missingness of any variables used in Model 1 (i.e., psychotic experiences, exposome variables, and age) and Model 2 (similar to Model 1 + family income and parental education) was 2,721 and 2,602 for males, and 2,401 and 2,310 for females, respectively. For clarification, we have noted the number of participants with family income data available in the footnote of Table 1 and also added the number of participants with missingness of any variables used in Model 1 and Model 2 in Table S1.

**1.3.12**  * Could the authors provide p-values when assessing associations? Despite the evident signals provided by odds ratio in clear association, the concepts might be misleading with regards to the reported "trend level".

**R1.3.12:** Thank you for pointing out this important issue. We have removed the term “trend level” and interpreted the result as not significant. However, due to the word limit, we only reported the 95% CIs of ORs, which represent both effect size and statistical significance, in the main text and provided p-values in the related tables. The revised text is shown below.

*“However, the isolated exposomic risk state (PRS-SCZ_75_=0 & ES-SCZ_75_=1) increased the odds of having past-month distressing PEs at a statistically significant level in females (OR 1.37 [95% CI 1.02, 1.85]), but not in males (OR 1.45 [95% CI 0.98, 2.16], Table 5). Similarly, the combined risk state (PRS-SCZ_75_=1 & ES-SCZ_75_=1) was associated with past-month distressing PEs significantly in females (OR 1.82 [95% CI 1.19, 2.77]) but not in males (OR 1.68 [95% CI 0.97, 2.90], Table 5).”*


**Reviewer #2:**

This study addresses the question of the impact of genetic and environmental risks for schizophrenia on psychotic experiences in adolescence and how these may differ by sex in a large well characterized cohort.

There are a few concerns which the authors may wish to address:

**2.1** A major concern is that the rationale for dichotomizing the PRS-SCZ and ES-SCZ at the 75th percentile is not clear. This is not standard practice and the reasons for the same need to be presented. It may be better to present analysis initially using them as continuous variables and then conducting sensitivity analyses using various cut-off points correcting appropriately for multiple testing.
**R2.1:** Thank you for your insightful suggestion. We have provided the rationale for dichotomizing the PRS-SCZ and ES-SCZ at the 75^th^ percentile as follows:

*“PRS-SCZ and ES-SCZ were dichotomized at the 75th percentile for each sex(hereafter PRS-SCZ_75_ and ES-SCZ_75_, respectively), at which cutoff has been shown to be associated with schizophrenia case-control status (Guloksuz et al. 2019), schizotypal traits in siblings and healthy participants (Pries et al. 2020), and PEs in this population (Di Vincenzo et al. 2025).”*

We have also conducted sensitivity analyses at various cutoff scores for PRS-SCZ and ES-SCZ and have provided the results in the Online Resource 2. The female-specific influences of PRS-SCZ (Figure S1) and universal effects of ES-SCZ (Figure S2) on PEs remained consistent. No significant additive interaction was detected at different combinations of PRS-SCZ and ES-SCZ cutoff scores (Table S6), similar to the main analysis.

**Figure S1** Associations between PRS-SCZ at different percentile cutoffs and PEs in males and females

**Figure S2** Associations between ES-SCZ different percentile cutoffs and PEs at in males and females

## **Table S6** Relative excess risk due to interaction (RERI) indicating additive interaction between genetic and exposomic risk states at different percentile cutoffs of PRS-SCZ and ES-SCZ on distressing PEs at 3-year follow-up in males and females

| Distressing PEs | PRS-SCZ cutoff | Males (N = 2,721) | | | Females (N = 2,401) | | |
| --- | --- | --- | --- | --- | --- | --- | --- |
|  |  | ES-SCZ cutoff | | | ES-SCZ cutoff | | |
|  |  | 30^th^ percentile | 60^th^ percentile | 90^th^ percentile | 30^th^ percentile | 60^th^ percentile | 90^th^ percentile |
| Past-month^c^  (3-year follow-up) | 30^th^ percentile | 0.23 [-0.99, 1.44] | 0.15 [-1.01, 1.31] | -0.79 [-2.44, 0.86] | 0.01 [-0.64, 0.66] | 0.04 [-0.63, 0.72] | -0.02 [-1.27, 1.24] |
|  | 60^th^ percentile | 0.08 [-1.02, 1.17] | 0.28 [-0.67, 1.22] | -0.47 [-1.64, 0.71] | -0.003 [-0.63, 0.63] | 0.34 [-0.26, 0.95] | 0.20 [-0.96, 1.36] |
|  | 90^th^ percentile | 1.19 [-1.02, 3.40] | 1.67 [-0.70, 4.04] | -0.93 [-3.04, 1.19] | 1.12 [-0.17, 2.40] | 1.33 [-0.26, 2.93] | -0.70 [-2.57, 1.17] |
| Lifetime  (≥ 1 wave) | 30^th^ percentile | 0.43 [-0.16, 1.02] | 0.53 [-0.33, 1.40] | 0.92 [-1.01, 2.86] | 0.54 [-0.35, 1.43] | 0.50 [-0.49, 1.49] | -0.23 [-4.48, 4.03] |
|  | 60^th^ percentile | 0.40 [-0.26, 1.06] | 0.27 [-0.66, 1.21] | 0.56 [-1.50, 2.62] | 0.50 [-0.49, 1.49] | 0.67 [-0.50, 1.85] | 2.01 [-2.16, 6.18] |
|  | 90^th^ percentile | -0.37 [-1.51, 0.77] | 0.12 [-1.32, 1.57] | -0.51 [-3.22, 2.20] | 0.92 [-1.16, 2.99] | 2.11 [-0.91, 5.13] | 35.3 [-37.7, 108.3] |
| Repeating  (≥ 2 waves) | 30^th^ percentile | 0.95 [0.07, 1.82] | 1.08 [-0.13, 2.28] | -0.04 [-3.16, 3.07] | 0.14 [-1.19, 1.47] | 0.45 [-0.82, 1.73] | 1.39 [-3.39, 6.17] |
|  | 60^th^ percentile | 1.00 [0.09, 1.91] | 1.03 [-0.17, 2.23] | 1.31 [-1.39, 4.01] | 0.45 [-0.82, 1.73] | 1.04 [-0.44, 2.51] | 4.20 [-1.71, 10.1] |
|  | 90^th^ percentile | 1.10 [-0.91, 3.10] | 0.23 [-2.08, 2.54] | -1.26 [-4.78, 2.25] | 1.20 [-1.63, 4.04] | 4.22 [-0.38, 8.82] | 26.6 [-18.0, 71.1] |
| Repeating  (≥ 3 waves) | 30^th^ percentile | 2.26 [-1.37, 5.90] | 2.62 [-1.64, 6.87] | 0.51 [-6.96, 7.99] | 0.36 [-1.20, 1.93] | 0.81 [-0.79, 2.40] | 2.09 [-1.59, 5.76] |
|  | 60^th^ percentile | 1.93 [-0.97, 4.84] | 1.35 [-2.17, 4.87] | 1.28 [-4.78, 7.34] | 0.81 [-0.79, 2.40] | 0.98 [-1.06, 3.03] | 3.36 [-1.40, 8.12] |
|  | 90^th^ percentile | 2.54 [-2.77, 7.85] | -0.08 [-5.67, 5.51] | 4.80 [-10.4, 20.0] | 3.84 [-0.27, 7.96] | 5.34 [-1.32, 12.00] | 15.4 [-11.6, 42.5] |
| Persisting  (all 4 waves) | 30^th^ percentile | -0.08 [-5.45, 5.29] | -0.25 [-6.26, 5.76] | 3.57 [-3.24, 10.4] | 0.53 [-5.04, 6.09] | -2.98 [-9.70, 3.75] | 3.16 [-5.39, 11.7] |
|  | 60^th^ percentile | 0.65 [-2.78, 4.08] | 0.11 [-4.23, 4.44] | -0.75 [-8.52, 7.02] | -2.98 [-9.70, 3.75] | 0.37 [-4.41, 5.15] | 3.55 [-5.10, 12.2] |
|  | 90^th^ percentile | -0.97 [-6.81, 4.87] | -1.69 [-7.45, 4.07] | NA | NA | NA | 8.25 [-18.1, 34.6] |

Note: All models were adjusted for age. No significant additive interactions were detected at any percentile cutoffs (Bonferroni-corrected *p* >.017). NA indicates a non-converged model.

**2.2** A decile plot may be used to describe the dose response relationship between PRS-SCZ and ES-SCZ and PEs, and would offer a good visualisation of the relationship across increasing levels of risk.
**R2.2:** Thank you for your insightful suggestion. We have provided the plots comparing ORs at different cutoff values of PRS-SCZ (Figure S1) and ES-SCZ (Figure S2), as shown in the preceding response.

**2.3** The additive interactions of PRS-SCZ and ES-SCZ on PEs may be non-significant due to inadequate power for the analysis.
**R2.3:** Thank you for your suggestion. We have now mentioned this in the limitation section as follows:

*“Lastly, our sex-stratified analyses reduced the sample size, possibly causing underpowered analyses*  *for low-prevalence outcomes (e.g., persisting distressing PEs) or for potentially weaker associations (e.g., interaction effects).”*

2.4 The discussion may comment upon the limitations of generalizability to non-European populations, the limitations of self-reported PEs compared to interview based PEs and the potential for reverse causality or residual confounding in the secondary outcome measures.

**R2.4:** Thank you for your thoughtful suggestions. We have discussed these points in the limitation section as shown:

*“First, we restricted the analysis to the European subsample to ensure good performance of PRS-SCZ based on recent GWAS, limiting generalizability to non-European populations. Second, PEs in the ABCD dataset were self-reported, potentially leading to false positives compared to interview-based assessments (Staines et al. 2023). However, prior evidence has shown that non-validated self-reported PEs are significantly associated with psychopathology similar to clinically validated PEs and attenuated psychosis syndrome (Moriyama et al. 2019). Furthermore, even false-positive self-reported PEs confirmed by a clinical interview appear to be associated with later mental health problems (van der Steen et al. 2019), supporting the clinical significance of our findings. Third, while genomic risks always precede outcomes, exposomic risks may have a bidirectional relationship with distressing PEs, potentially inflating associations. To mitigate this, we adjusted for prior-wave distressing PEs in past-month analyses, reducing the likelihood of overestimating ES-SCZ effects. However, this approach could not be applied to secondary outcomes, leaving the possibility of reverse causality or residual confounding* *for the analysis of ES-SCZ.”*

**References:**

Di Vincenzo, M, Prachason, T, Sampogna, G, Arias-Magnasco, A, Lin, BD, Pries, LK, van Os, J, Rutten, BPF, Barzilay, R, Fiorillo, A, & Guloksuz, S (2025) Independent and joint effects of genomic and exposomic loads for schizophrenia on psychotic experiences in adolescents of european ancestry. *Schizophrenia (Heidelb)*, *11*(1), 26. <https://doi.org/10.1038/s41537-025-00569-2>

Guloksuz, S, Pries, LK, Delespaul, P, Kenis, G, Luykx, JJ, Lin, BD, Richards, AL, Akdede, B, Binbay, T, Altinyazar, V, Yalincetin, B, Gumus-Akay, G, Cihan, B, Soygur, H, Ulas, H, Cankurtaran, E, Kaymak, SU, Mihaljevic, MM, Petrovic, SA, . . . van Os, J (2019) Examining the independent and joint effects of molecular genetic liability and environmental exposures in schizophrenia: Results from the eugei study. *World Psychiatry*, *18*(2), 173-182. <https://doi.org/10.1002/wps.20629>

Lam, M, Awasthi, S, Watson, HJ, Goldstein, J, Panagiotaropoulou, G, Trubetskoy, V, Karlsson, R, Frei, O, Fan, CC, De Witte, W, Mota, NR, Mullins, N, Brugger, K, Lee, SH, Wray, NR, Skarabis, N, Huang, H, Neale, B, Daly, MJ, . . . Ripke, S (2020) Ricopili: Rapid imputation for consortias pipeline. *Bioinformatics*, *36*(3), 930-933. <https://doi.org/10.1093/bioinformatics/btz633>

Pries, LK, Dal Ferro, GA, van Os, J, Delespaul, P, Kenis, G, Lin, BD, Luykx, JJ, Richards, AL, Akdede, B, Binbay, T, Altinyazar, V, Yalincetin, B, Gumus-Akay, G, Cihan, B, Soygur, H, Ulas, H, Sahin Cankurtaran, E, Ulusoy Kaymak, S, Mihaljevic, MM, . . . Guloksuz, S (2020) Examining the independent and joint effects of genomic and exposomic liabilities for schizophrenia across the psychosis spectrum. *Epidemiol Psychiatr Sci*, *29*, e182. <https://doi.org/10.1017/S2045796020000943>

Pries, LK, Lage-Castellanos, A, Delespaul, P, Kenis, G, Luykx, JJ, Lin, BD, Richards, AL, Akdede, B, Binbay, T, Altinyazar, V, Yalincetin, B, Gumus-Akay, G, Cihan, B, Soygur, H, Ulas, H, Cankurtaran, ES, Kaymak, SU, Mihaljevic, MM, Petrovic, SA, . . . Guloksuz, S (2019) Estimating exposome score for schizophrenia using predictive modeling approach in two independent samples: The results from the eugei study. *Schizophr Bull*, *45*(5), 960-965. <https://doi.org/10.1093/schbul/sbz054>
